# An Intron-Retaining Splice Variant of Human Cyclin A2, Expressed in Adult Differentiated Tissues, Induces a G1/S Cell Cycle Arrest In Vitro

**DOI:** 10.1371/journal.pone.0039249

**Published:** 2012-06-20

**Authors:** Arata Honda, Yannick Valogne, Myriam Bou Nader, Christian Bréchot, Jamila Faivre

**Affiliations:** 1 Tokyo Metropolitan Health and Medical Treatment Corporation, Ebara Hospital, Tokyo, Japan; 2 INSERM, U785, Centre Hépatobiliaire, Villejuif, France; 3 Université Paris-Sud, Faculté de Médecine, Villejuif, France; Virginia Commonwealth University, United States of America

## Abstract

**Background:**

Human cyclin A2 is a key regulator of S phase progression and entry into mitosis. Alternative splice variants of the G1 and mitotic cyclins have been shown to interfere with full-length cyclin functions to modulate cell cycle progression and are therefore likely to play a role in differentiation or oncogenesis. The alternative splicing of human cyclin A2 has not yet been studied.

**Methodology/Principal Findings:**

Sequence-specific primers were designed to amplify various exon–intron regions of cyclin A2 mRNA in cell lines and human tissues. Intron retaining PCR products were cloned and sequenced and then overexpressed in HeLa cells. The subcellular localization of the splice variants was studied using confocal and time-lapse microscopy, and their impact on the cell cycle by flow cytometry, immunoblotting and histone H1 kinase activity. We found a splice variant of cyclin A2 mRNA called A2V6 that partly retains Intron 6. The gene expression pattern of A2V6 mRNA in human tissues was noticeably different from that of wild-type cyclin A2 (A2WT) mRNA. It was lower in proliferating fetal tissues and stronger in some differentiated adult tissues, especially, heart. In transfected HeLa cells, A2V6 localized exclusively in the cytoplasm whereas A2WT accumulated in the nucleus. We show that A2V6 induced a clear G1/S cell cycle arrest associated with a p21 and p27 upregulation and an inhibition of retinoblastoma protein phosphorylation. Like A2WT, A2V6 bound CDK2, but the A2V6/CDK2 complex did not phosphorylate histone H1.

**Conclusion/Significance:**

This study has revealed that some highly differentiated human tissues express an intron-retaining cyclin A2 mRNA that induced a G1/S block in vitro. Contrary to full-length cyclin A2, which regulates cell proliferation, the A2V6 splice variant might play a role in regulating nondividing cell states such as terminal differentiation or senescence.

## Introduction

Cyclins play an essential role in progression through the eukaryotic cell cycle, acting as regulatory subunits of cyclin-dependent kinases (CDKs). Types A and B cyclins are more specifically responsible for the onset of mitosis, and through their degradation, the exit from mitosis. Their activities are determined by changes in their subcellular localization during successive phases of the cell cycle [1].

Cyclin A2 achieves its regulatory activity predominantly, if not exclusively, in the nucleus from the G1/S transition to mitosis, participating in the entry into, and progress through, S phase, DNA replication, centrosome duplication, and the entry into mitosis [2]–[4]. The existence of cytoplasmic cyclin A2 in the physiologic situation has long been controversial. However, several reports have described the presence of cyclin A2 within the cytoplasm during the S phase [5], [6] and within the centrosome during mitosis [7], [8]. Cyclin A/CDK2 complexes have been found in the microsomal and endocytic compartments of regenerative liver cells [9]–[11]. It has previously been reported that endoplasmic reticulum-associated non-degraded cyclin A2 was able to interact with, and activate, CDKs [12] and had the ability to transform *in vivo* and *in vitro* [13]. Traffic of cyclin A2/CDK2 complexes between the nucleus and cytoplasm is a dynamic process [14], and an isoform of cyclin A2 exists in the cytoplasm [15], [16]. The biological role of cytoplasmic cyclin A2 remains to be elucidated.

Alternative splicing can generate multiple transcripts encoding proteins with subtle or opposing functional differences that can have profound biological consequences [17]. Many alternative splicing events occur only in a specific tissue at a specific time during development and/or under certain physiological conditions [17]. Previous estimates, based on analysis of expressed sequence tags (EST), suggest that the transcripts from 35% of human genes are alternatively spliced [18], [19]. Several forms of cyclin E mRNA were detected and cloned [20], [21]. Some were expressed in tumor tissues and not in non-tumor tissues [22], [23], and revealed alternate substrate affinity [24]. A splice variant of cyclin D1 encodes a protein with an altered C-terminal domain and has been detected in a range of cell lines and tissues. It is localized constitutively in the nucleus and is a potent oncogenic agent [25]. Lozano *et al.* reported a splice variant of cyclin B with retention of an intronic sequence, which was first discovered in a sea urchin and observed to be abundant in the oocyte of the embryo [26]. This variant differs from wild-type cyclin B in the structure of the C-terminal and may be involved in the control of cell division and differentiation. The same group subsequently reported the existence of splicing variants of human cyclin B3 [27].

Based on the homology of the C-terminal sequences of cyclins B and A, we hypothesized the existence of splice variants of cyclin A2. In this study, we identified and analyzed a splice variant of cyclin A2, termed A2V6, which retains Intron 6. A2V6 is highly expressed in adult tissues such as the heart, liver, and kidney but is not expressed in the same tissues in the fetus. We demonstrated that A2V6 was localized exclusively in the cytoplasm of transfected HeLa cells. Furthermore, it induced the G1/S cell-cycle block and bound CDK2 without stimulating its histone H1-kinase activity. This suggests that A2V6 may play a role in the regulation of cellular differentiation.

## Results

### An Intron-retaining Cyclin A2 Splice Variant is Expressed in Human Tissues

**Figure 1:**
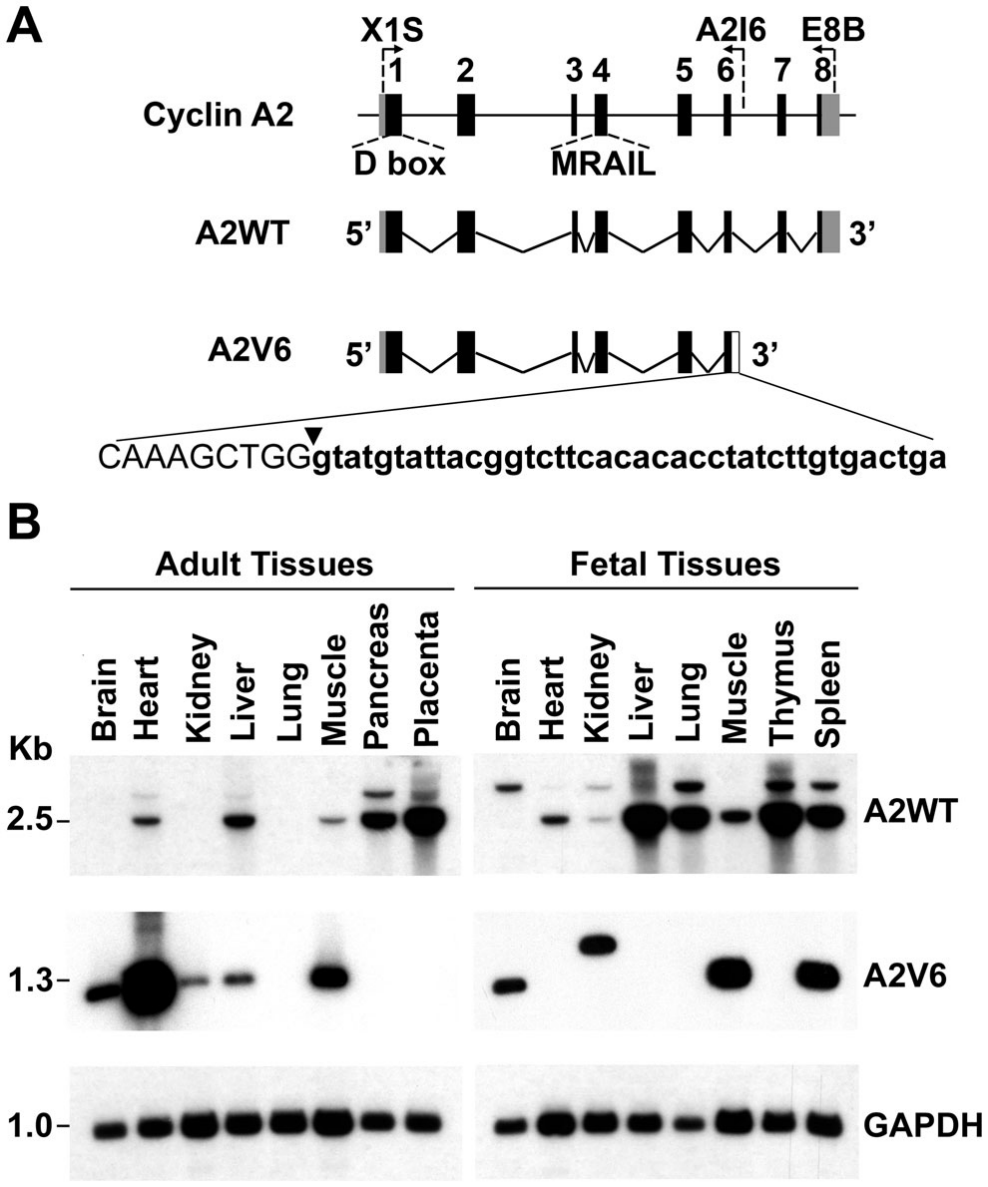
Distribution of the A2V6 splice variant of cyclin A2 in human tissues. (**A**) Exon-Intron organization of the Human cyclin A2 gene. Black boxes: exons. Grey boxes: 5′ and 3′ UTR. D-box: destruction box. MRAIL: CDK2 binding motif belonging to the cyclin box. Cyclin A2: gene. A2WT: mature cyclin A2 messenger resulting from alternate RNA splicing and amplified using the X1S-E8B primer pair. A2V6: cyclin A2 splice variant mRNA retaining a portion of Intron 6 (empty box) amplified using the X1S-A2I6 primer pair. The indicated sequence comprises the last nine nucleotides of Exon 6 (uppercase letters) and the retained Intron 6 sequence (lowercase letters). (**B**) Gene-expression analysis of cyclin A2 using PCR-Southern blotting in human adult and fetal tissue samples from the indicated organs. A2WT: wild-type cyclin A2 mRNA (2.5 kb). The 3.3 kb PCR product was determined to be A2WT plus Intron-1 by sequencing. A2V6: cyclin A2 splice variant (1.3 kb). GAPDH: Glyceraldehyde-3-phosphate dehydrogenase (loading control).

B-type cyclins are subject to an alternative splicing that gives rise to C-terminus intron retention [26]. Knowing that A- and B-type cyclins have large regions of homology in their C terminus, we sought intron-retaining splice variants of cyclin A2 in human adult and fetal tissues. The human cyclin A2 gene is organized in 8 exons displaying canonical intron/exon and exon/intron borders (Fig. 1A). The length of the mature cyclin A2 (A2WT) mRNA is of 2.5 kb. Using sequence-specific primers designed to amplify exon–intron regions, we were able to amplify a 1.3 kb cDNA in some human tissues (brain, kidney, muscle, heart, spleen, liver), which was showed by sequencing to comprise the first six exons and a retained portion (GTATGTATTACGGTCTTCACACACCTATCTTGTGAC) of Intron 6 of cyclin A2 (Fig. 1A and B). Gene-expression analysis by PCR-Southern blotting revealed preferentially the 2.5 kb A2WT mRNA in highly proliferating fetal and placental tissues (Fig. 1B), consistent with A2WT being a S phase cyclin [28], [29]. It is interesting to note that the expression of A2V6 mRNA was noticeably lower than that of A2WT mRNA in the proliferating fetal tissues. In contrast, A2V6 mRNA was more strongly detected than A2WT mRNA in some differentiated adult tissues, namely, the brain, kidney, muscle, and, especially, heart. It should be noted that fetal kidney displayed a signal of higher molecular weight (1.5 kb) than A2V6 mRNA. This signal was found, by sequencing, to correspond to an mRNA splice variant containing Exons 1 to 6 and Introns 5 and 6. These observations establish the existence of a tissue-specific alternative splicing of the mRNA of cyclin A2, and suggest a link between cyclin A2 alternative splicing and tissue differentiation.

### Cyclin A2V6 Variant Protein is Sequestered in the Cytoplasm During the Cell Cycle

**Figure 2:**
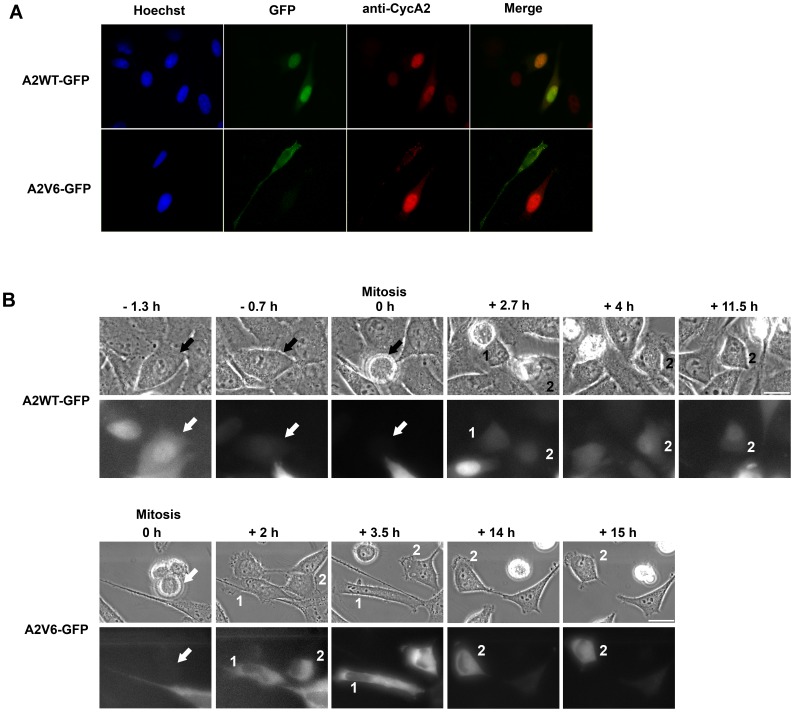
Cytoplasmic localization of A2V6-GFP during the cell cycle. HeLa cells were transfected with wild-type cyclin A2-GFP (A2WT-GFP) or Intron 6-retaining cyclin A2-GFP (A2V6-GFP) vectors. (**A**) Immunofluorescence images of transfected HeLa cells using anti-GFP (green) and anti-cyclin A2 (red) antibodies. Nuclei (blue) were counterstained with Hoechst dye 33258. The A2V6-GFP protein is located in the cytoplasm of transfected cells. No endogenous cyclin A2 is detected in cells expressing the A2V6 protein. (**B**) Time-lapse imaging of A2WT- and A2V6-GFP during a cell cycle. Upper panel: Phase contrast microscopy. Lower panel: Fluorescence microscopy. Arrow: parent cell. Labels 1 and 2: daughter cells. Wild-type cyclin A2-GFP is accumulated in G2 nuclei (−1.3 h), degraded in mitosis and reappears mostly in the nuclei in S phase, like endogenous cyclin A2. In contrast, A2V6-GFP remains in the cytoplasm throughout the cell cycle. Scale bar: 20 µm.

To gain insight into the biological role of cyclin A2 splice variants, we investigated the subcellular localization of the A2WT-GFP and A2V6-GFP fusion proteins in HeLa cells. We used GFP tags to trace those cells, which overexpress the corresponding constructs, and an anti-cyclin A2 antibody, which recognizes endogenous cyclin A2, exogenous A2WT-GFP, and, with a lesser efficacy, exogenous A2V6-GFP. A hundred of cells transfected by each construct were examined. We found that the A2V6-GFP fusion protein localized exclusively in the cytoplasm of transfected cells, contrary to A2WT-GFP, which was predominantly located in the nucleus (Fig. 2A). Anti-cyclin A2 immunodetection showed that all the examined A2WT-GFP transfected cells had a nuclear cyclin A2 staining. About 20% of the non-transfected cells displayed such a staining. None of the hundred A2V6-GFP transfected cells studied displayed any nuclear endogenous cyclin A2, suggesting that these cells do not undergo S phase. Staining with specific organelle markers showed that A2V6-GFP did not localize in early or late endosomes, Golgi apparatus, or the endoplasmic reticulum (data not shown). Next, the dynamics of A2WT-GFP and A2V6-GFP was followed in mitotic and daughter transfected HeLa cells by time-lapse imaging. Figure 2B shows that A2WT-GFP was concentrated in the nuclei in S/G2, degraded during mitosis (time point 0 h), re-detected in the daughter cells during G1 (2.7 h), and finally translocated again into the nuclei (4 h and 11.5 h), which is similar to the behavior of endogenous cyclin A2 [30], [31]. In contrast, A2V6-GFP remained accumulated in the cytoplasm of transfected cells during the whole cell cycle. Notably, A2V6-GFP was not translocated to the nuclei in S Phase. Therefore, the A2V6-GFP expressing cells did not progress through the cell cycle and rested in G1 until apoptosis.

### Cyclin A2v6 Splice Variant Induces G1/S Cell Cycle Arrest

**Figure 3:**
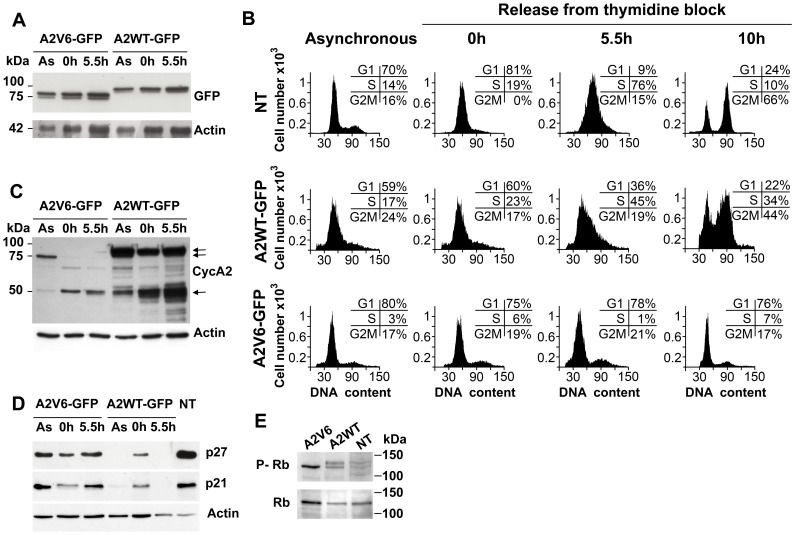
Overexpression of A2V6-GFP induces a G1/S arrest. HeLa cells were transfected with wild-type cyclin A2-GFP (A2WT-GFP) or Intron 6-retaining cyclin A2-GFP (A2V6-GFP) vectors. The analyses were performed in asynchronous cells (As), in thymidine-synchronized cells (0 h) and at the indicated times (5.5 h, 10 h) after thymidine removal. (**A**) Anti-GFP immunoblot. The molecular mass of the GFP fusion proteins is 75–80 kDa. Actin: normalization using β-actin. (**B**) Cell cycle analysis using flow cytometry. Inset: percentage of cells in the different phases of the cell cycle. NT: non-transfected cells. Cells expressing A2V6-GFP were arrested in G1/S phase while cells expressing endogenous cyclin A2 or A2WT-GFP were progressing through the cell cycle. (**C**) Anti-cyclin A2 immunoblot in lysates from cells expressing A2V6- and A2WT-GFP showing the expression of endogenous cyclin A2 (single arrow) and the GFP fusion proteins (double arrow). Actin: normalization using β-actin. (**D**) Immunoblots for the CDK inhibitors p21 and p27 in lysates from cells expressing A2V6- and A2WT-GFP demonstrating their accumulation in A2V6-GFP expressing cells (lane 5.5 h). NT: non-transfected cells. (**E**) Anti-total-retinoblastoma (Rb) and anti phospho-Rb immunoblots showing that phosphorylated Rb was present in A2WT-GFP and non-transfected (NT) cell extracts, and not in A2V6-GFP cell extracts.

We investigated the effect of the A2V6 splice variant on the cell cycle by FACS analysis of HeLa cells synchronized at the G1/S border using double thymidine block. The expression levels of A2V6-GFP were similar to those of A2WT-GFP in asynchronous and synchronized cell cultures, as revealed by anti-GFP immunoblotting (Fig. 3A). FACS analysis of asynchronous cell cultures revealed a substantial difference in cell cycle distribution between A2V6- and A2WT-GFP-expressing cells, suggesting that A2V6 does not play the same role as A2WT in cell cycle progression (Fig. 3B). Thymidine synchronization allowed us to compare the activities of the two fusion proteins in S entry and progression. A2WT-GFP-expressing cells had moved from G1/S to the S phase by 5.5 h after thymidine removal (Th5.5) and a large proportion of them had completed a cell cycle at Th10. In contrast, most (76±2%) of the A2V6-GFP-expressing cells were not capable of progressing through the cell cycle and rested in G1/S up to Th10 (Fig. 3B). Next, to gain insight into the biochemical underpinnings of prevention of the G1/S transition, we investigated the expression of ectopic and endogenous cyclin A2 in cells transfected with A2WT-GFP and A2V6-GFP constructs. Anti-cyclin A2 immunoblots showed that endogenous cyclin A2 was hardly expressed in A2V6-GFP-transfected cells, whereas its expression level was strongly increased in A2WT-GFP-transfected cells 5.5 h after thymidine-block release (Fig. 3C). This is consistent with the G1/S arrest of A2V6-GFP-transfected cells and the noticeable progress into S phase of A2WT-GFP-transfected cells observed by flow cytometry upon release from the thymidine block. It is known that the negative regulators of the cell cycle p21 and p27 are downregulated during the G1 to S-phase progression allowing the cyclin A2-associated kinase activity to be deployed [32], [33]. We found that both p21 and p27 were indeed suppressed in A2WT-GFP-transfected cells at Th5.5, but were on the contrary accumulated in A2V6-GFP-expressing cells, in agreement with the cell cycle arrest that we have found in these cells (Fig. 3D). We also know that the progression of cells into S phase is associated with the hyperphosphorylation of the Retinoblastoma protein (Rb) releasing the E2F transcription factor, which in turn transactivates proliferative genes [34]–[37]. Immunoblotting for phospho-Rb displayed the hyperphosphorylated form of Rb in total cell lysates from A2WT-GFP-transfected cells, but not those from A2V6-GFP-expressing cells (Fig. 3E).

### The Cyclin A2V6 Splice Variant Forms an Inactive Complex with CDK2

**Figure 4:**
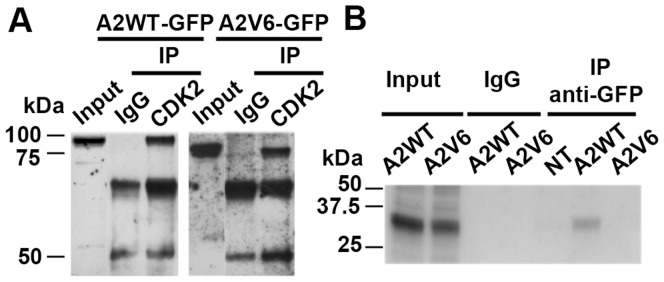
A2V6-GFP binds CDK2 without inducing H1 kinase activity. (**A**) Anti-GFP immunoblot (IB) from cell lysates immunoprecipitated (IP) with anti-CDK2 antibody. The A2V6-GFP fusion protein (≈75 kDa) interacts with CDK2 like the A2WT-GFP fusion protein (≈85 kDa). IgG: non-specific antibody control. (**B**) Autoradiography of ^32^P-labeled histone H1 performed after anti-GFP immunoprecipitation in lysates from A2WT- and A2V6-GFP transfected cells. IgG: non-specific antibody control. Input: non-immunoprecipitated lysate. No phosphorylated histone H1 was detected in A2V6-GFP transfected cells, indicating that the A2V6/CDK2 complexes, contrary to the A2WT/CDK2 ones, are not functional. The phosphorylated histone H1 in the input lanes corresponds to the global kinase activity of cycling asynchronous cells.

Cyclin A2 is known to form complexes with the protein kinase CDK2 and thereby phosphorylate key substrates to promote S phase and DNA replication [38]. Anti-CDK2 immunoprecipitation followed by anti-GFP immunoblotting in lysates from asynchronous A2WT- and A2V6-GFP-transfected cells showed bands at about 85 and 75 kDa, respectively, corresponding to the molecular size of the fusion proteins. This showed that the binding efficiency of the A2V6 variant protein to CDK2 is comparable to that of A2WT (Fig. 4A). Histone H1 kinase assays revealed that the A2V6/CDK2 complex, contrary to A2WT/CDK2, did not phosphorylate histone H1 in vitro (Fig. 4B).

## Discussion

Evidence concerning the role of cyclins D and E in human carcinogenesis has accumulated [39], [40]. In contrast, no evidence of the transforming capacity of cyclin A2 has yet been provided. However, a previous study based on an HBV-cyclin A2 fusion protein suggested a new mechanism of cyclin-related cell transformation [41]. Furthermore, it has been demonstrated that an endoplasmic reticulum (ER)-associated cyclin A2 mutant triggers CDK activation [12], over-duplicates centrosome, increases ploidy, and transforms cells [13]. These findings suggested that the truncated forms of cyclin A2 has a different behaviour from that of full-length cyclin A2, and is perhaps involved in cell transformation.

This study has demonstrated the presence of an intron-retaining cyclin A2 mRNA in some highly differentiated human tissues, such as the heart, liver, and kidney. Its expression in adult heart tissue was particularly marked. Cyclin A2 is generally found in proliferating somatic tissues and is normally silenced in the heart shortly after birth when cardiomyocyte division ceases [42], [43]. Our findings suggest a role for the A2V6 splice variant mRNA in cell differentiation through regulation of G1 phase.

This study emphasizes the existence of an endogenous A2V6 mRNA in human cells. Even though specific antibodies were raised against the peptide specific of the retained intronic sequence, no endogenous A2V6 protein could be detected. We therefore overexpressed A2V6 protein in HeLa cells and studied its subcellular localization during the cell cycle, and its effect on cell-cycle progression.

Cyclin A2 is localized predominantly within the nucleus [15]. The synthesis of cyclin A2 is initiated in the late G1 phase, and a degradation of cyclin A2 protein by the anaphase-promoting complex (APC) occurs during prophase [3]. Interestingly, we found that A2V6 was localized exclusively in the cytoplasm and thereby had a critical effect on cell-cycle progression. An overexpressed cytoplasmic A2V6 splice variant induced a long-duration G1/S arrest following thymidine-block release, which could lead to cell apoptosis. Cytoplasmic localization of endogenous cyclin A2 has long been controversial. It has been reported that a N-terminal-truncated cyclin A isoform was localized in the cytoplasm and bound CDK2, suggesting that the nuclear localization signal (NLS) of cyclin A2 is located in its N-terminal part [44]. There are reports of N-terminal-truncated mutants of cyclin A2 without destruction box (D-box) that were located in the cytoplasm, escaped degradation and possessed transforming activity [13], [41]. In this study, the A2V6 splice variant contains the N-terminal part upstream of Exon 6, which includes NLS and D-box, and lacks the C-terminal part downstream of Exon 7. The fact that, nevertheless, A2V6 was non-degraded until apoptosis could be a direct consequence of the G1/S cell-cycle block.

Importantly, FACS analysis revealed that A2V6 has an inhibitory effect on G1/S transition as a result of its sequestration in the cytoplasm. Prevention of the G1/S transition related to A2V6 was associated with an upregulation of p21 and p27 and an absence of the hyperphosphorylated form of Rb, which suppressed the expression of endogenous cyclin A2 and therefore hindered cell cycle progression. This could be due to A2V6 occupying the CDK2 binding site as an agonist and thus competing with A2WT. Finally, the complex that was formed by A2V6 and CDK2 did not induce histone H1 kinase activity. This suggests that the C-terminal-truncated A2V6 is rendered kinase defective by some conformational change, while the N-terminal part, which includes the binding CDK domain, remains able to interact with CDK2 [45]. Additionally, it should be noted that unknown cytoplasmic substrates might be phosphorylated by A2V6-associated kinase, which would give a molecular basis to the G1/S arrest induced by A2V6.

In conclusion, we have identified a cyclin A2 splice variant mRNA with intronic retention called A2V6 that is preferentially expressed in some human adult tissues and have demonstrated a G1/S arrest in A2V6-transfected HeLa cells. This suggests that endogenous A2V6 may play a role in the regulation of irreversible nondividing cell states such as terminal differentiation or senescence. Moreover, the fact that A2V6 induces cell growth arrest and apoptosis designates this molecule as a potential anticancer molecule. Further studies will be necessary to substantiate these conclusions.

## Materials and Methods

### RNA Extraction and RT-PCR

Total RNA extraction from HeLa cell (ATCC CCL-2) lysates was performed using TRIzol Reagent (Gibco BRL). The lysate was mixed with 200 µL chloroform for 30 s at room temperature (RT) for 3 min, centrifuged for 15 min at 12,000 g and 4°C. The RNA is then precipitated from the aqueous phase in 500 µL isopropanol. The RNA pellet was washed with 1 mL ethanol 75%, centrifuged for 5 min at 7,500 g and 4°C, dried and resuspended in 10 µL of RNA-free water. The mRNA was isolated by magnetic separation using Dynabeads Oligo (dT) (Dynal) and reverse transcribed with Superscript Transcriptase (Gibco BRL) and gene specific primer for Exon 8 of human cyclin A2 (E8B: nt. 10102-10127, 5′-TACTCAACACTTATAGAGGTTTGCTC-3′). The cDNA was PCR amplified with oligonucleotide primers designed for splice variant (A2V6) and wild type of cyclin A2 (A2WT). Sense primer was located in Exon 1 and anti-sense primer in Intron 6 or Exon 8. The primer sequences were: sense primer, X1S (nt. 2972-2993, 5′-GGCGGCTACGACTATTCTTTGG-3′); anti-sense primer, A2I6B (nt. 7837-7860, 5′-GCACAGGCATTTCAGTCACAAGAT-3′); anti-sense primer, E8B. Five micrograms of cDNA from cells and tissue panels (Clontech) were amplified using Advantage cDNA polymerase kit (Clontech) for 35 cycles of 94°C for 30 s, 60°C for 90 s, and 72°C for 90 s.

### Southern Hybridization

RT-PCR products separated on 1% agarose gel were transferred to a positively charged nylon membrane (GE Healthcare) by the method of Southern. Oligonucleotide probes were labeled with [^32^P]dCTP using Megaprime DNA Labeling System (GE Healthcare). Hybridization was performed in Church buffer [28]. Briefly, membranes were prehybridized for 1 hour at 65°C and then hybridized in the same buffer with 1×10^6^ cpm/mL of labeled probe overnight at 65°C. After hybridization, each filter was washed twice with 2X SSC solution, twice with 0.2X SSC solution, and then exposed to X-ray film.

### Plasmid Constructs

The PCR fragments X1S-A2I6B (A2V6) and X1S-E8B (A2WT) were ligated in the pGEM-T cloning vector (Promega) and the the JM109 competent bacteria were transformed. Positive clones were selected by PCR with T7 primer and gene specific human cyclin A2 primer X4R (nt. 6018–6042, 5′-CCACAAGCTGAAGTTTTCCTCTCAG-3′) for 25 cycles of 94°C for 45 s, 60°C for 45 s, and 72°C for 45 s. The restriction EcoRI/NotI fragment was separated on a 10% agarose gel, purified by QIA quick gel purification kit (Qiagen) and inserted into the pEGFP-C1 vector (Clontech) to create the pEGFP-A2V6 and pEGFP-A2WT vectors, and into pcDNA3.1/Myc-His (Invitrogen) to create the pMyc-His-A2V6 and pMyc-His-A2WT vectors.

### Cell Culture, Transient Transfection, and Synchronization

HeLa cells were cultured in Dulbecco’s MEM with GlutaMAX containing 10% fetal bovine serum (FBS) at 37°C and 5% CO2. Cells grown on glass coverslip were transfected using ExGen 500 transfection reagent (Euromedex). Briefly, cells were plated at 1.5×10^5^ cells per well in 6-well plates. Twenty-four hours later, 1 mL of serum-free medium containing 8 µL of the ExGen 500 reagent and 2 µg of plasmid DNA was added to each well for 24 h. G1/S cell synchronization was done using double thymidine block. The cells were treated with the 2 mM thymidine for 17 h then changed to fresh medium for 9 h and replaced with 2 mM thymidine medium for 15 h.

### Immunofluorescence Confocal Microscopy and Time-lapse Videomicroscopy

The transfected cells were fixed in 3% formaldehyde, rinsed and permeabilized in phosphated-buffered saline (PBS) containing 0.2% Saponine. Fixed cells were incubated with the primary antibodies for 45 min at RT and washed with PBS. They were then incubated with Cy3- and Cy5-conjugated secondary antibodies for 45 min at RT. Cell nuclei were stained with 4′,6-diamino-2-phenylindole (DAPI, Sigma). Sequential images were taken at 0.4 µm intervals using a SP2 AOBS confocal microscope (Leica). Time-lapse videomicroscopy was performed using a digital microscope (Leica) equipped with a high-resolution CCD camera (CoolSnap HQ, Photometrix) and a high-sensitivity camera (Pentamax) driven by a Metamorph 6 software (Universal Imaging).

### Flow Cytometric Analysis

GFP-expressing cells were detached with trypsin-EDTA and washed twice with ice-cold PBS, and 1×110^6^ cells were resuspended in 500 µL of cold PBS. The cellular suspension was mixed with 10 µg/mL Hoechst 33342 (1 mg/mL) and incubated at 37°C for 45 min. Analysis by flow cytometry was performed collecting 20,000 events per sample on a FACSCalibur flow cytometer (BD Biosciences) using the CellQuest software (BD Biosciences).

### Immunoblotting and Immunoprecipitation

At 48 h post-transfection, total protein extracts were prepared with a buffer containing 30 mM Tris HCl pH 7.5, 250 mM NaCl, 0.5% NP40, 1 mM sodium orthovanadate, 1 mM NaF and protease inhibitors. For immunoprecipitation assays, cleared lysates were incubated with anti-CDK2 antibody. Equal amounts of extracts (40 µg) were separated on 8% SDS-PAGE, transferred onto nitrocellulose (GE Healthcare) and processed for immunoblotting. Incubations were performed with a lysis buffer containing 20 mM Tris-HCl pH 7.5, 137 mM NaCl, 0.05% Tween 20 and 5% nonfat dry milk. The HRP-conjugated secondary antibodies were used at a dilution of 1∶2000 (GE Healthcare). The chemoluminescence ECL Plus reagent was purchased from GE Healthcare.

### In vitro Kinase Reaction

Anti-GFP immunoprecipitates were washed three times with kinase buffer (50 mM Tris HCl pH 7.5, 20 mM EGTA, 10 mM MgCl2, 1 mM DTT, 1mM β-glycerolphosphate), and then incubated in 50 µL of kinase reaction buffer with 100 µM cold ATP and 10 µCi [γ-^32^P]ATP for 30 min at 25°C. Samples were analyzed by SDS-PAGE followed by autoradiography.

### Antibodies

The antibodies used were: Mouse anti-c-Myc monoclonal (9E10) and rabbit anti-CDK2 polyclonal (M2) antibodies (Santa Cruz Biotechnology); Mouse anti-GFP monoclonal (JL-8) antibody (Clontech); Mouse anti-human cyclin A2 monoclonal (11B2G3) antibody (Immunotech); Rabbit anti-p21 polyclonal antibody (Santa Cruz Biotechnology); Rabbit anti-p27 polyclonal antibody (Santa Cruz Biotechnology); Rabbit anti-pRb polyclonal antibody (Cell Signaling).
